# Is Caring for Grandchildren Good for Grandparents’ Health? Evidence From a Fourteen-wave Nationwide Survey in Japan

**DOI:** 10.2188/jea.JE20200529

**Published:** 2022-08-05

**Authors:** Takashi Oshio

**Affiliations:** Institute of Economic Research, Hitotsubashi University, Tokyo, Japan

**Keywords:** grandchildren, grandparents, psychological distress, self-rated health

## Abstract

**Background:**

Enhanced female labor force participation is raising the importance of grandparents’ caring for their grandchildren. However, previous studies have reported mixed results of the association between grandchild care and grandparents’ health.

**Methods:**

Longitudinal data of 33,204 individuals born between 1946 and 1955 were collected from a 14-wave nationwide panel survey conducted from 2005 to 2018. We examined how caring for at least one co-residing grandchild aged <6 years was associated with grandparents’ psychological distress (defined by five or higher Kessler 6 score) and poor self-rated health in pooled cross-sectional, fixed-effects, and 3-year follow-up logistic models.

**Results:**

While pooled cross-sectional models showed a positive association between grandchild care and grandparents’ health, the fixed-effects or follow-up logistic models did not find any significant association between them. In the case of grandmothers, the odds ratio of reporting psychological distress in response to caring for grandchildren was 0.98 (95% confidence interval [CI], 0.89–1.08) and 1.04 (95% CI, 0.85–1.27) observed from fixed-effects and 3-year follow-up models, respectively, compared to 0.86 (95% CI, 0.81–0.91) in the pooled cross-sectional model. Similar patterns were observed for self-rated health for grandmothers, while grandfathers’ health outcomes were not sensitive to grandchild care. These results contrasted with those of caring for parents, which had almost consistently a negative association with grandparents’ health.

**Conclusion:**

The results suggest that caring for grandchildren does not have a beneficial or detrimental effect on grandparents’ health.

## INTRODUCTION

Grandparents tend to play a vital role in caring for grandchildren, especially in the intergenerational family setting, wherein multigenerational co-residence is considered a social norm.^1^^–^^4^ If grandparents reside with their children, they may be involved in grandchild care on a daily basis and thus promote filial piety and family solidarity, especially in traditional Asian societies.^5^ In a more recent context, grandparents’ involvement in childbearing has become increasingly important considering a rising trend of labor force participation by married women and an increase in single parent families.^6^^–^^8^

However, the impact of grandchild care on grandparents’ health is difficult to predict. Grandchild care may enhance self-esteem, self-worth, or family cohesion, and accordingly have a beneficial impact on health.^9^^–^^11^ More broadly, providing care to grandchildren is expected to make grandparents stay active and maintain their health status from deteriorating at older ages.^12^ However, grandchild care may be physically and psychologically demanding for grandparents.^13^^–^^15^ Indeed, studies have provided mixed evidence of the association between grandchild care and grandparents’ health.^2^ Some studies showed positive associations,^9^^,^^11^^,^^13^^,^^16^^–^^20^ while others reported negative or null correlations.^14^^,^^15^^,^^21^^–^^25^

One plausible reason for the mixed results is a difference in the analytic strategy applied in the statistical analysis, besides a difference in the intensity of care^24^ and socio-cultural backgrounds.^26^ Generally, cross-sectional analysis tended to show a favorable health impact of grandchild care.^11^^,^^13^^,^^16^^–^^18^^,^^20^ However, their results may be biased by reverse causality and/or simultaneity biases. We cannot exclude the possibility that healthier grandparents are more likely to care for their grandchildren. A grandparent’s certain attributes may also simultaneously affect care and health, leading to a spurious correlation.

Meanwhile, longitudinal studies have tended to show negative or mixed health effects.^14^^,^^15^^,^^21^^–^^25^ Specifically, prospective cohort analysis, which focuses on grandchild care in a baseline year (or its change from a baseline year to a follow-up year) on health outcomes in a follow-up year, has often been conducted to mitigate these biases. Such an analysis tended to show less beneficial health effects of care or even their absence.^14^^,^^15^^,^^21^^,^^23^^–^^25^ However, neither cross-sectional nor prospective cohort analysis can fully control for an individual’s unobserved attributes, which may cause biased estimations. Fixed-effects model analysis, which can control for an individual’s time-invariant attributes, is expected to attenuate such biases.^22^^,^^27^

We attempted to examine the association between grandchild care and grandparents’ health using data obtained from a population-based, 14-wave survey in Japan. This study is expected to add to the relevant literature in two ways. First, we examined how the results would depend on the choice of statistical approaches: pooled cross-sectional, fixed-effects, and prospective cohort models. Fixed-effect models, which control for an individual’s time-invariant attributes, concentrate on variations within each individual.^27^ For the prospective cohort model, we focused on grandparents who cared for their grandchildren for 3 continuous years to examine the health impact of continuous caring for grandchildren. We also controlled for the potential attrition bias by applying the inverse probability weighting method.^28^^,^^29^

Second, we compared the health effects of caring for grandchildren and that of parents. Many studies have provided evidence of the negative impact of caring for elderly parents on their informal caregivers regardless of the kinship relations between caregivers and receivers.^30^^–^^34^ However, few studies have compared it with that of caring for grandchildren using the same dataset and a consistent analytic framework. We further examined whether caring for both grandchildren and parents would have any additional effect on caregivers’ health. A recent study using Chinese data showed that such “sandwich” caregivers reported greater subjective well-being.^35^ Furthermore, we compared the results between grandmothers and grandfathers. Previous studies have provided mixed results of gender differences in the health impact of grandchild care, probably reflecting a difference in the intensity of care or roles played in the family.^6^^,^^9^^,^^35^

The findings of this study are expected to provide new insights into the health of middle-aged and older adults in Japan. While the proportion of three-generation households out of total households consistently declined from 15.3% in 1986 to 5.1% in 2019,^36^ rising trends of labor force participation, an increase in single parent families, and the limited availability of formal nursing services are likely to raise attention to the role of grandparents’ caring for grandchildren and its health implications.

## METHODS

### Study sample

In this study, we used data obtained from a nationwide 14-wave panel survey, “The Longitudinal Survey of Middle-Aged and Older Adults,” conducted by the Japanese Ministry of Health, Labour and Welfare (MHLW) each year from 2005 to 2016. Japan’s Statistics Law required the survey to be reviewed from statistical, legal, ethical, and other viewpoints. We obtained the survey data from the MHLW with its official permission; therefore, the current study did not require ethical approval.

**Figure 1.**
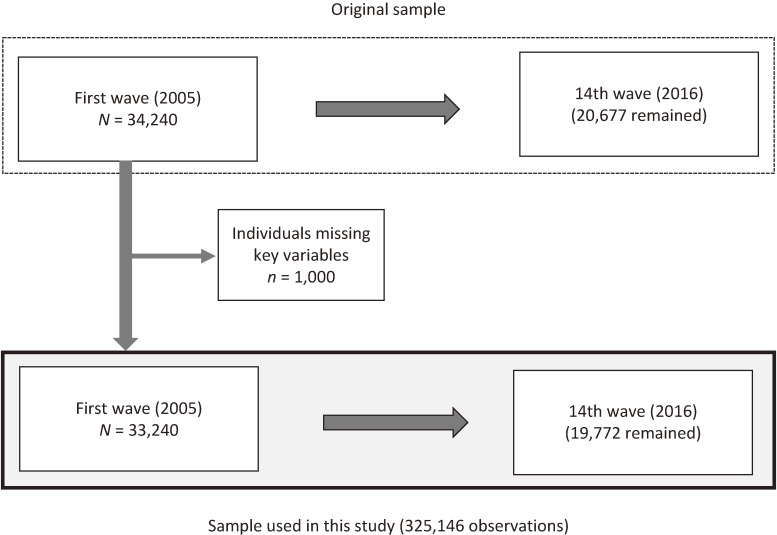
Structure of the sample used in this study

The survey started with the cohort aged 50–59 years (born in 1946 to 1955) in the first wave. A total of 34,240 individuals responded (response rate: 83.8%). The second to fourteenth waves of the survey were conducted each year from 2006 to 2018 and 20,677 individuals remained in the 14th wave. We used the longitudinal data of 33,240 individuals after excluding those missing key variables (Figure 1). We used 325,146 observations of these individuals over the entire 14 waves.

### Measures

#### Caring for grandchildren and parents

We first constructed a binary variable of caring for grandchildren by allocating one to the respondents who were caring for at least one co-residing grandchild aged 5 years or less, and zero to others. This was aimed at focusing on grandparents’ custodial, rather than occasional, caring for co-residing grandchildren. The survey asked the respondent whether they were caring for their children *or* grandchildren, making it difficult to distinguish caring for grandchildren from that for children. To address this, we removed the respondents who had at least one co-residing child who was younger than 20 years from the study sample. Furthermore, we constructed a binary variable of caring for parents by allocating one to the respondents who reported that they were caring for at least one of their parents and parents-in-law and zero to others.

We also constructed binary variables of long-term caring for grandchildren and parents, respectively, by allocating one to those who had been caring for them for 3 continuous years and one to others (including those who had cared once or twice during the past 3 years). We focused on 3 continuous years, which were close to the average duration of caring for each of the grandchildren and parents, as shown later in Table 1. We limited the analysis to the respondents who were found to have started (or resumed) caring 2 years earlier to the survey year and kept caring for 3 subsequent years.

**Table 1.** Key features of the study sample

		All	Women	Men
Educational attainment (%)				
Junior high school		17.9	17.5	18.4
High school		54.0	58.7	48.8
Junior college		6.8	10.9	2.3
College		14.6	6.2	23.7
Other and unknown		6.8	6.7	6.8

Features at baseline				
Having a spouse, %		83.7	82.7	84.8
Having a paid job, %		78.9	67.2	91.6
Age, years	*M*	54.7	54.7	54.8
	*SD*	(2.7)	(2.7)	(2.7)
Household spending,^a^ thousand JPY; monthly	*M*	186.9	180.9	193.3
	*SD*	(192.1)	(162.6)	(219.2)

Experience during 14 waves				
Caring for grandchildren, %		10.9	12.7	9.0
Starting age, years	*M*	55.2	55.7	54.9
	*SD*	(4.4)	(4.6)	(4.2)
Duration, years	*M*	3.5	3.6	3.2
	*SD*	(2.6)	(2.6)	(2.4)
Caring for parents, %		28.4	30.4	26.2
Starting age, years	*M*	58.1	57.7	58.7
	*SD*	(4.3)	(4.1)	(4.3)
Duration, years	*M*	3.1	3.3	2.9
	*SD*	(2.6)	(2.7)	(2.4)
Caring for both grandchildren and parents, %		1.6	1.9	1.2
Starting age, years	*M*	53.5	53.5	53.5
	*SD*	(3.4)	(3.7)	(3.2)
Duration, years	*M*	1.8	1.9	1.7
	*SD*	(1.3)	(1.4)	(1.1)

*N*		33,240	17,304	15,936

^a^Household-size adjusted.

#### Health outcomes

We considered two binary variables of health outcomes: psychological distress and poor self-rated health (SRH). We measured psychological distress using K6 scores.^37^^,^^38^ Earlier studies have confirmed the reliability and validity of this score in psychological analyses of Japanese people.^39^^,^^40^ The respondents were asked to answer a six-item psychological distress questionnaire—“During the past 30 days, about how often did you feel a) nervous, b) hopeless, c) restless or fidgety, d) so depressed that nothing could cheer you up, e) that everything was an effort, and f) worthless?”—rated on a 5-point scale (0 = *none of the time* to 4 = *all of the time*). Further, the sum of the reported scores (range: 0–24) was calculated and defined as the K6 score. Higher K6 scores reflect higher levels of psychological distress. Cronbach’s alpha was 0.897 for the entire study sample. K6 scores = 5+ indicate mood/anxiety disorder in a Japanese sample, as validated by preceding studies.^37^^,^^39^ We constructed a binary variable of psychological distress by allocating one to K6 scores = 5+ and zero to others. Regarding SRH, the respondents were asked to choose 1 (*very good*), 2 (*good*), 3 (*somewhat good*), 4 (*somewhat poor*), 5 (*poor*), or 6 (*very poor*) regarding their current health condition. SRH has been found to be correlated with morbidity and predictive of changes in functional ability; thus, it can serve as a global measure of health status in the general population.^41^^,^^42^ We constructed a binary variable of poor SRH by allocating one to those who chose 4, 5, or 6, and zero to others.

#### Covariates

We considered a set of covariates: age, educational attainment (junior high school, high school, junior college, college or above, and others and unanswered), having a spouse, having a paid job, and current smoking. As a proxy for household income, we further considered household spending adjusted for household size by dividing it by the square root of the number of household members.^43^ We categorized it into quartiles and constructed binary variables for each quartile and unanswered. We also controlled for health variables: psychological distress, SRH, and whether there were any problems in activities of daily living (ADL) at baseline.

### Statistical analysis

We estimated three types of logistic regression models: pooled cross-sectional, fixed-effects, and 3-year follow-up models, to explain the probability of psychological distress or poor SRH by caring for grandchildren and/or parents as well as a set of covariates.

In fixed-effects models, an individual’s time-invariant attributes, both observed and unobserved, were removed from the regression analysis. We also conducted the Hausman test to test the null hypothesis that an individual’s time-invariant attributes are not correlated with dependent variables.^27^

For 3-year follow-up models, we considered whether caring for grandchildren or parents for 3 continuous years was associated with the probability of reporting psychological distress or poor SRH. In this analysis, we controlled for health variables (psychological distress, SRH, and whether having any ADL problem) at baseline as well as other covariates. To attenuate the attrition biases, we applied the inverse probability method.^28^^,^^29^ To this end, we first estimated the probit model to explain the probability that a respondent would stay in the survey until the fourth wave conditional on the participation in the baseline, using a respondent’s attributes observed in the baseline. Subsequently, we used the inverse of the estimated probability as a weight in the regression model to predict the health impact of 3-year caring.

We estimated these regression models for women and men. For all statistical analyses, we used the software package Stata (Release 15; STATA Corp, College Station, TX, USA).

## RESULTS

Table 1 summarizes the key features of the participants in the survey. As seen in this table, 12.7% and 9.0% of women and men, respectively, experienced caring for grandchildren during 14 waves, starting at 55.7 and 54.9 years on average. The prevalence of caring for parents was much higher: 30.4% and 26.2% for women and men, respectively, and it started somewhat later at 57.7 and 58.7 years old, respectively. The average duration of caring for grandchildren was 3.6 and 3.2 years for women and men, respectively, somewhat longer than caring for parents (3.3 and 2.9 years). Simultaneous caring for both grandchildren and parents was relatively rare, with a prevalence of less than 2% for both women and men.

For the descriptive analysis, Table 2 shows how the prevalence of psychological distress and poor SRH differs across caregivers and non-caregivers among women and men, respectively. Among women, the prevalence of psychological distress was somewhat lower among caregivers for grandchildren (but not parents) compared to non-caregivers: 27.3% compared to 29.8% (difference: *P* < 0.001). The same was true for poor SRH (*P* < 0.001). Similar patterns were observed among men, but the difference in SRH was not significant. In contrast, the proportion of caregivers for parents reporting psychological distress or poor SRH was much higher than non-caregivers among both women and men (*P* < 0.001).

**Table 2.** Difference in prevalence of psychological distress and poor self-rated health between caregivers and non-caregivers

%	Proportion	Psychological distress	Poor self-rated health	*N*
Women				
All	100.0	30.7	18.8	175,719
Neither caring for grandchildren or parents	86.9	29.8	18.5	152,747
Caring for grandchildren but not parents	4.0	27.3	17.4	6,980
Caring for grandparents but not grandchildren	8.8	41.6	22.9	15,388
Caring for both grandchildren and parents	0.3	45.0	23.2	604
Caring for grandchildren for 3 years	0.5	31.1	18.6	791
Caring for parents for 3 years	0.8	42.2	22.9	1,455

Men				
All	100.0	25.8	20.5	149,427
Neither caring for grandchildren or parents	90.0	25.3	20.4	134,457
Caring for grandchildren but not parents	2.8	23.9	19.5	4,170
Caring for grandparents but not grandchildren	7.0	34.0	22.8	10,465
Caring for both grandchildren and parents	0.2	29.6	23.3	335
Caring for grandchildren for 3 years	0.3	26.3	21.9	494
Caring for parents for 3 years	0.7	32.8	22.7	1,043

We further noticed two things from this table. First, caring for both grandchildren and parents made the proportions of reporting psychological distress and poor SRH higher than caring for only grandchildren (*P* < 0.05) among both men and women, but did not so compared to caring for only parents. Second, caring for grandchildren for 3 continuous years did not significantly affect the proportion of reporting psychological distress or poor SRH compared to no care provision among both women and men. By comparison, 3-year continuous caring for parents raised the proportion of reporting psychological distress and poor SRH among both women and men (*P* < 0.001).

Table 3 and Table 4 report the key results of pooled cross-sectional, fixed-effects, and 3-year follow-up models for women and men, respectively. We confirmed that the Hausman tests showed that fixed-effects models were preferred to random-effects models in all cases.

**Table 3.** Estimated associations of cares for grandchildren and parents with health for women^a^

	Pooled cross-sectional			Fixed-effects			3-year follow up^b^		

	OR		95% CI	OR		95% CI	OR		95% CI
Psychological distress									
Caring for grandchildren	0.86	^***^	(0.82, 0.91)	0.98		(0.89, 1.08)			
Caring for parents	1.66	^***^	(1.61, 1.72)	1.68	^***^	(1.58, 1.79)			
Caring for grandchildren and parents	1.31	^***^	(1.10, 1.56)	1.16		(0.89, 1.51)			
Caring for grandchildren for 3 years							1.04		(0.85, 1.27)
Caring for parents for 3 years							1.58	^***^	(1.38, 1.81)

*N*	175,719			107,004			106,118		

Poor self-rated health									
Caring for grandchildren	0.89	^***^	(0.84, 0.96)	1.09		(0.97, 1.22)			
Caring for parents	1.38	^***^	(1.33, 1.44)	1.37	^***^	(1.28, 1.48)			
Caring for grandchildren and parents	1.11		(0.91, 1.37)	0.89		(0.65, 1.21)			
Caring for grandchildren for 3 years							0.99		(0.77, 1.27)
Caring for parents for 3 years							1.39	^***^	(1.19, 1.63)

*N*	175,719			86,055			106,118		

CI, confidence interval; OR, odds ratio.

^a^Controlled for covariates (educational attainment, having a spouse, having a paid job, household spending, and current smoking).

^b^Inverse probability weighted Health variables (psychological distress, self-rated health, and whether there are any problems in activities of daily living) in the baseline were additionally controlled for.

^***^P < 0.001, ^**^P < 0.01, ^*^P < 0.05.

**Table 4.** Estimated associations of cares for grandchildren and parents with health for men^a^

	Pooled cross-sectional			Fixed-effects			3-year follow up^b^		

	OR		95% CI	OR		95% CI	OR		95% CI
Psychological distress									
Caring for grandchildren	0.95		(0.88, 1.02)	1.03		(0.91, 1.17)			
Caring for parents	1.51	^***^	(1.45, 1.58)	1.53	^***^	(1.42, 1.64)			
Caring for grandchildren and parents	0.90		(0.70, 1.15)	0.75		(0.51, 1.08)			
Caring for grandchildren for 3 years							1.26		(0.98, 1.64)
Caring for parents for 3 years							1.40	^***^	(1.19, 1.66)

*N*	149,427			83,682			91,505		

Poor self-rated health									
Caring for grandchildren	0.95		(0.88, 1.03)	0.98		(0.85, 1.12)			
Caring for parents	1.17	^***^	(1.11, 1.23)	1.22	^***^	(1.12, 1.32)			
Caring for grandchildren and parents	1.08		(0.83, 1.42)	0.76		(0.50, 1.15)			
Caring for grandchildren for 3 years							1.22		(0.91, 1.64)
Caring for parents for 3 years							1.16		(0.97, 1.38)

*N*	149,427			74,461			91,505		

CI, confidence interval; OR, odds ratio.

^a^Controlled for covariates (educational attainment, having a spouse, having a paid job, household spending, and current smoking).

^b^Inverse probability weighted Health variables (psychological distress, self-rated health, and whether there are any problems in activities of daily living) in the baseline were additionally controlled for.

^***^P < 0.001, ^**^P < 0.01, ^*^P < 0.05.

The results of the pooled cross-sectional models were consistent with the descriptive analysis. Specifically, the odds ratio (OR) of reporting psychological distress in response to caring for grandchildren was 0.86 (95% confidence interval [CI], 0.82–0.91), suggesting a favorable health impact of grandchild care. This contrasts with caring for parents with an OR of 1.66 (95% CI, 1.61–1.72). We also observed increased odds of caring for grandchildren and parents (OR 1.31; 95% CI, 1.10–1.56), suggesting that the double care amplified psychological distress.

In contrast, the fixed-effects and follow-up models showed that the ORs of reporting psychological distress in response to caring for grandchildren were 0.98 (95% CI, 0.89–1.08) and 1.04 (95% CI, 0.85–1.27), respectively, indicating no significant association between grandchild care and psychological distress. Unlike caring for grandchildren, a positive association between caring for parents and psychological distress was shown even by the fixed-effects (OR 1.68; 95% CI, 1.58–1.79) and follow-up models (OR 1.58; 95% CI, 1.38–1.81). We also found that simultaneous caring for both grandchildren and parents did not add to psychological distress in the fixed-effects model (OR 1.16; 95% CI, 0.89–1.51).

Similar results were observed for poor SRH. Fixed-effects and follow-up models showed that grandchild care was not correlated with the probability of reporting poor SRH, with OR 1.09 (95% CI, 0.97–1.22) and OR 0.99 (95% CI, 0.77–1.27), respectively, while the pooled cross-sectional model showed that it was negatively associated with that probability (OR 0.89; 95% CI, 0.84–0.96). Caring for parents consistently had a negative association with SRH. The interaction between care for grandchildren and parents had no additional impact on SRH (OR 0.89; 95% CI, 0.65–1.21).

Table 4 reports the regression results for men. The most notable difference from the results for women is that grandchild care was not significantly related with psychological distress (OR 0.95; 95% CI, 0.88–1.02) or SRH (OR 0.95; 95% CI, 0.88–1.03) even in the pooled cross-sectional models. We also made two other observations: (1) caring for parents had consistently a negative association with SRH, and (2) caring for both grandchildren and parents did not amplify their association with SRH, which remained the same as that for women.

## DISCUSSION

We examined how grandparents’ health was associated with caring for grandchildren using a nationwide longitudinal survey in Japan. The results suggest that grandchild care does not have a beneficial or detrimental effect on grandparents’ health. Unlike the descriptive analysis and pooled cross-sectional regression models, fixed-effects or 3-year follow-up regression models did not indicate any significant association between them. Meanwhile, grandfathers’ health outcomes were not sensitive to grandchild care, even in the pooled cross-sectional regression models.

A combination of (1) a positive association between grandchild care and health observed from the pooled cross-sectional analysis and (2) a non-significant association between them observed from the fixed-effects and 3-year follow-up models are consistent with a general pattern observed from previous cross-sectional^13^^,^^16^^–^^19^^,^^21^ and longitudinal studies.^14^^,^^15^^,^^22^^–^^26^

One possible explanation for the gap between the observations from cross-sectional and longitudinal analyses is that certain unobserved (time-invariant) attributes of an individual may make grandparents more inclined to be selected into grandchild care, and simultaneously, make them feel healthier. For instance, individuals with higher neuroticism may be more reluctant to care for their grandchildren and simultaneously be more inclined to assess their health negatively. Meanwhile, we cannot argue that a positive relationship between grandchild care and health observed from the cross-sectional data suggests that healthier individuals are more likely to be selected as grandchild caregivers. If such a reverse causation exists, fixed-effects results would have shown a positive association between caring for grandchildren and health.^22^ Moreover, the results from the 3-year follow-up models did not show any significant association between continuous caregiving for grandchildren and grandparents’ health.

Thus, we did not find any significant association between caring for grandchildren and grandparents’ health. This sharply contrasts with caring for parents, which almost consistently had a negative association, as already shown by many studies.^30^^–^^33^

One possible interpretation for the lack of a significant association between grandchild care and grandparents’ health may be that favorable and unfavorable impacts of grandchild care on health were largely offset from each other. A positive association of caring for grandchildren on health observed from cross-sectional data, even if overstated, may point to a favorable aspect of grandchild care for health, probably reflecting an enhanced feeling of family cohesion, life satisfaction, and other subjective well-being.^1^^–^^5^ However, no significant association observed from longitudinal analyses in this study suggests that such a favorable impact of grandchild care on health may be largely offset by its adverse impact on health, probably due to its physically and psychologically demanding aspects. In the case of care for parents, we can argue that its negative impact on health dominates the positive one even under the longitudinal data setting. It should also be noted that grandfathers’ health was generally insensitive to grandchild care, presumably reflecting their lower intensity of care compared with grandmothers.

This study has several limitations. First, it did not precisely identify the causation from care to health. The fixed-effects models controlled for an individual’s time-invariant attributes, but this does not mean they identified the causality. The 3-year follow-up models incorporated the time gap between care and health and controlled for prior health, but they could not exclude the case that a change in health affected a change in care. Second, we did not consider the intensity of grandchild care or domains of care provision, due to limited information available from the dataset. As mentioned above, the insensitivity of grandfathers’ health outcomes to grandchild care may be attributable to their lower intensity of care. Differences in the domains of care provision are also likely to cause gender differences in the health impact of caregiving. Third, we need more analysis to understand the mechanism that links grandchild care and grandparents’ health, an issue not addressed in this study. This mechanism can also be confounded by grandparents’ roles in family, other social activities, and their relations with neighbors and friends. Fourth, we should be cautious of any generalization of the observed results. The results may depend heavily on socio-cultural contexts, which is another potential reason for mixed results in previous studies.^26^ Specifically, a beneficial impact on grandparents’ health from grandchild care, presumably via enhanced self-esteem, self-worth, or family cohesion, may be closely linked to the social norm regarding intergenerational family setting, which may influence the role expected to be played by grandparents.^9^^–^^11^

Despite these limitations, the results suggest that caring for grandchildren had no beneficial or detrimental impact on grandparents’ health. This contrasts with caring for patients, which is another major life event for middle-aged individuals and tends to have an adverse impact on health. A more detailed longitudinal analysis on the dynamics of health outcomes of grandchild care is needed, considering that grandchild care is closely related to family arrangement and female labor force participation.
